# Supplementary material for: Unraveling the varied nature and roles of defects in hybrid halide perovskites with time-resolved photoemission electron microscopy
Source: Energy Environ Sci. 2021 Sep 15;14(12):6320–8. doi: 10.1039/d1ee02055b (PMC8658252; doi:10.1039/d1ee02055b)
Supplement: EE-014-D1EE02055B-s001 [file EE-014-D1EE02055B-s001.pdf]

Electronic Supplementary Material (ESI) for Energy & Environmental Science.
This journal is © The Royal Society of Chemistry 2021

Electronic Supplementary Information (ESI†)

for Energy & Environmental Science

Unraveling the varied nature and roles of defects in hybrid halide perovskites with time-resolved photoemission electron microscopy

Sofiia Kosar[a], Andrew J. Winchester[a], Tiarnan A. S. Doherty[b], Stuart Macpherson[b], Christopher E. Petoukhoff[a], Kyle Frohna[b], Miguel Anaya[b,c], Nicholas S. Chan[a], Julien Madéo[a], Michael K. L. Man[a], Samuel D. Stranks*[b,c], Keshav M. Dani*[a]

[a] Femtosecond Spectroscopy Unit, Okinawa Institute of Science and Technology, 1919-1 Tancha, Onna-son, Okinawa, 904-0495, Japan. E-mail: kmdani@oist.jp

[b] Cavendish Laboratory, University of Cambridge, JJ Thomson Avenue, Cambridge CB3 0HE, United Kingdom. E-mail: sds65@cam.ac.uk

[c] Department of Chemical Engineering and Biotechnology, University of Cambridge, Philippa Fawcett Drive, Cambridge CB3 0AS, United Kingdom.

Fig. S1 Photoluminescence spectroscopy for perovskite thin film sample.

Fig. S2 Characterization of perovskite films with and without of gold fiducial markers.

Fig. S3 Image registration nXRD/PEEM.

Fig. S4 Spatially averaged x-ray diffraction pattern.

Fig. S5 Energy-resolved PEEM and size distribution of defect clusters.

Fig. S6 Additional photoemission spectra and work function maps.

Fig. S7 Additional TR-PEEM images and traces.

Fig. S8 Additional regions with photoluminescence (PL) maps around defect clusters (PEEM), and SEM images for selected defects.

Fig. S9 Additional photoemission spectra and work function maps after treatment with dry air and light.

Fig. S10 TR-PEEM response of polytype and PbI_2 defect clusters upon exposure to light and dry air.

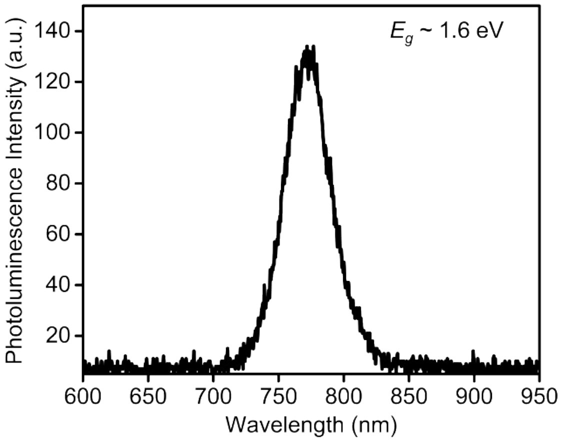

Fig. S1 Photoluminescence spectroscopy for perovskite thin film sample. PL spectrum with 532 nm excitation.

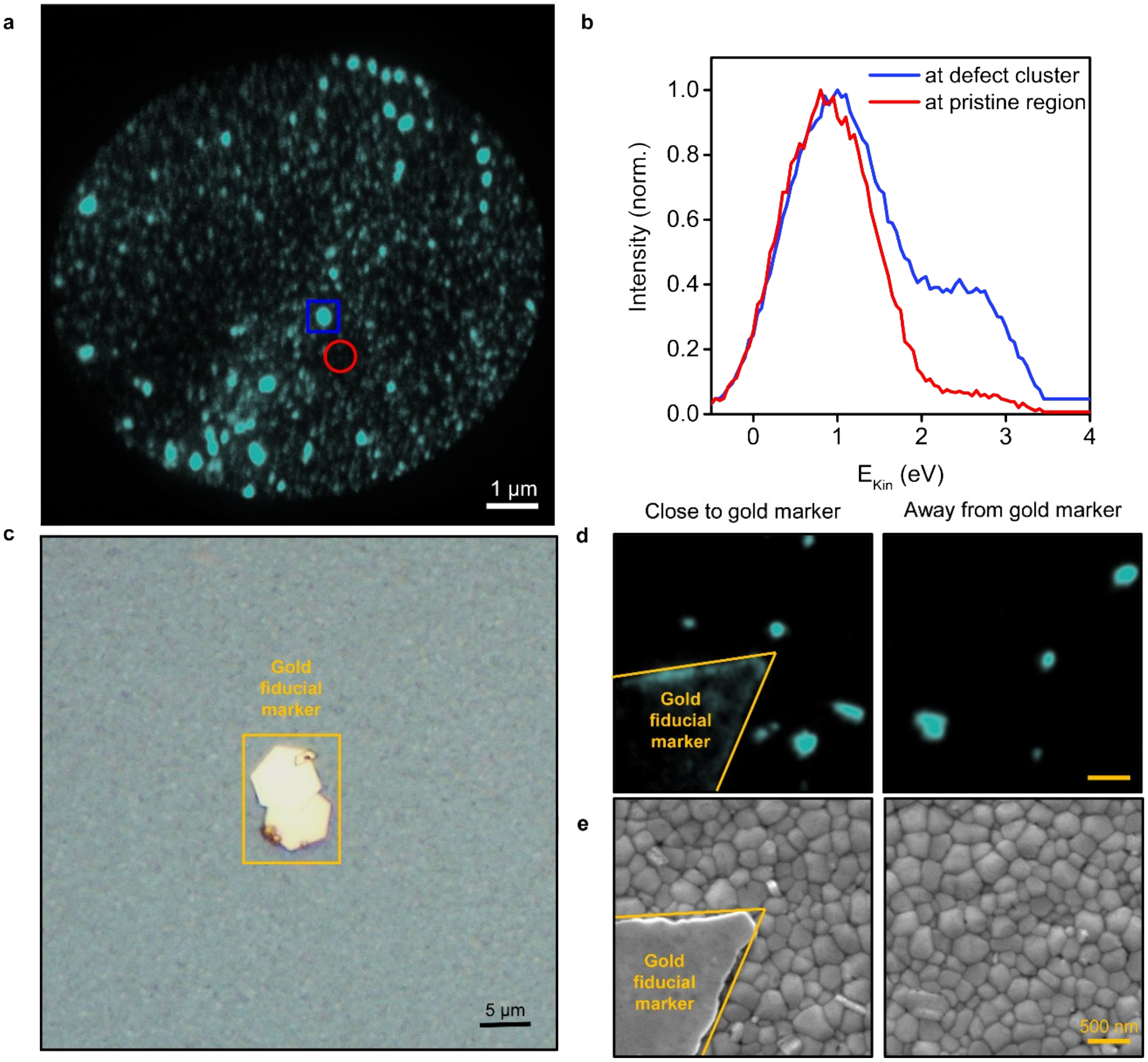

Fig. S2 Characterization of perovskite films with and without of gold fiducial markers. (a) PEEM image with 4.6 eV photons, showing defect distribution for sample without gold markers. (b) Photoelectron spectra (PES) extracted from defect cluster (blue square in (a) and pristine area (red circle in (a)). (c) Optical microscopy image of a typical gold fiducial marker deposited on perovskite film. No visible changes to the film coverage close to and away from the marker observed. (d) PEEM images taken with 4.65 eV probe photons in close proximity and away from gold fiducial marker revealing similar distribution of defect clusters within these regions. (e) SEM images for the same regions provided in (d), showing no alteration to the surface morphology in the regions close to gold markers.

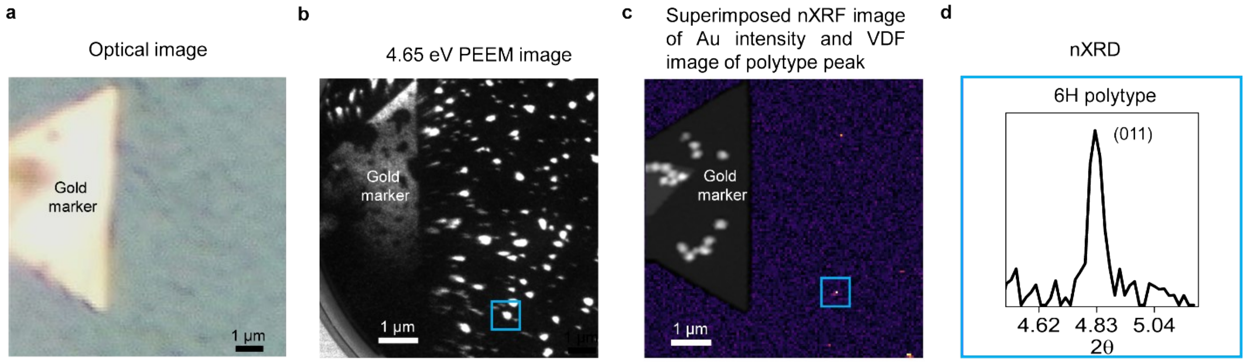

Fig. S3 Image registration nXRD/PEEM. (a) Optical image of region of interest with gold fiducial marker. (b) 4.65 eV PEEM image of region of interest revealing defect clusters and gold marker. (c) Superimposed nXRF image of Au intensity and Virtual Dark Field (VDF) image of polytype peak within the region of interest revealing shape of gold marker and spatial location showing (011) peak of 6H hexagonal perovskite polytype. The XRF image clearly shows the gold marker and was used for image registration with the PEEM map. (d) Local diffraction pattern extracted from the defect cluster marked with a blue box in (b) and (c) showing the (011) peak of a 6H hexagonal perovskite polytype.

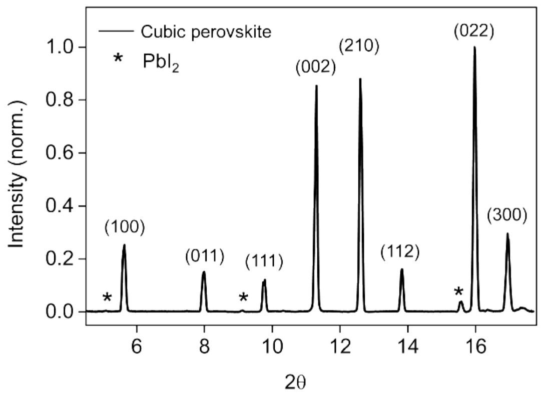

Fig. S4 Spatially averaged x-ray diffraction pattern. Extracted from the same region as shown in Fig. 1c, indexed to a cubic perovskite structure, with detected PbI_2 peaks.

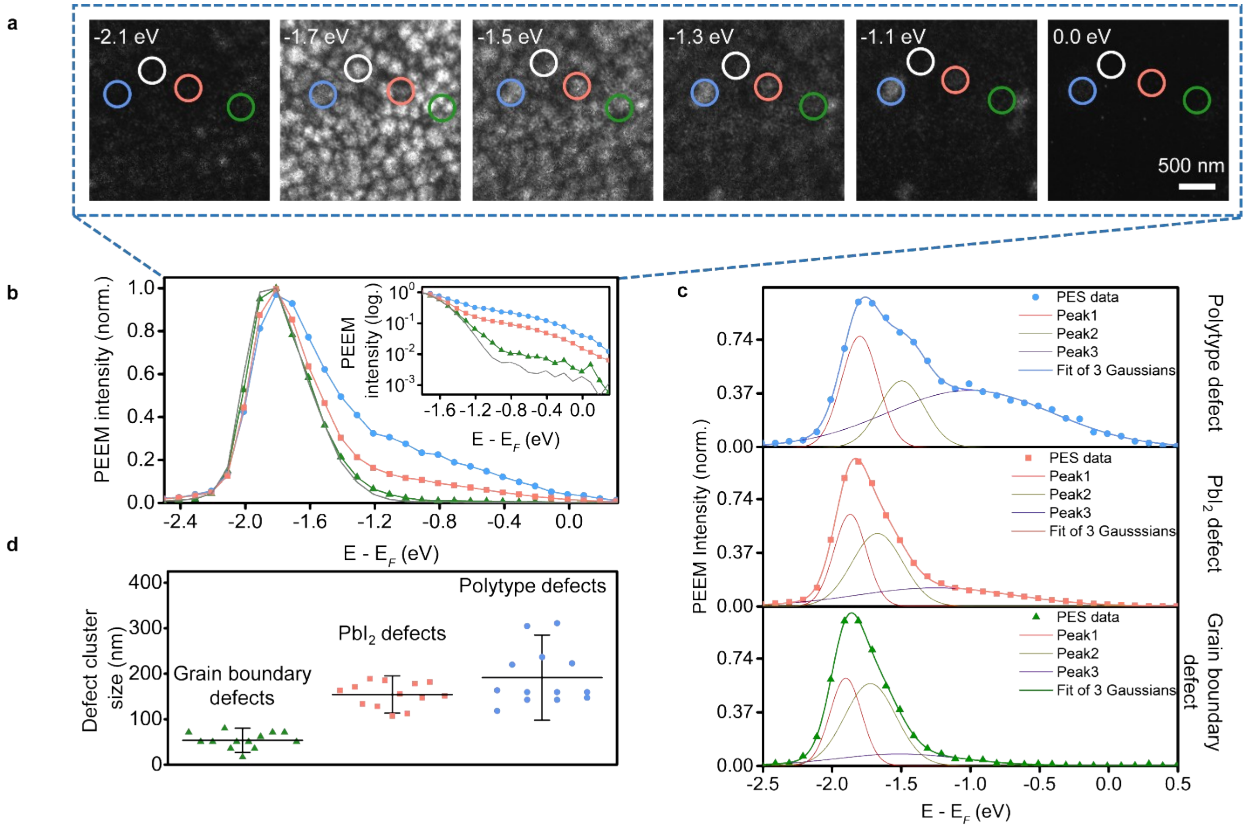

Fig. S5 Energy-resolved PEEM and size distribution of defect clusters. (a) Representative set of energy-resolved PEEM images showing surface morphology at (~ - 1.7 eV) and appearance of localized defect states as clusters at lower energies. (b) Photoelectron spectra for three types of defect clusters, averaged for multiple clusters of the same type, and region without defects (grey solid line). (c) Gaussian fits (red, yellow, purple) to the photoelectron spectra for selected defect clusters used to estimate peak energy of the mid-gap defect states for three types of defect clusters. (d) Size distribution for three types of defect clusters, as labelled; solid black line indicates the mean value, error bars show standard deviation.

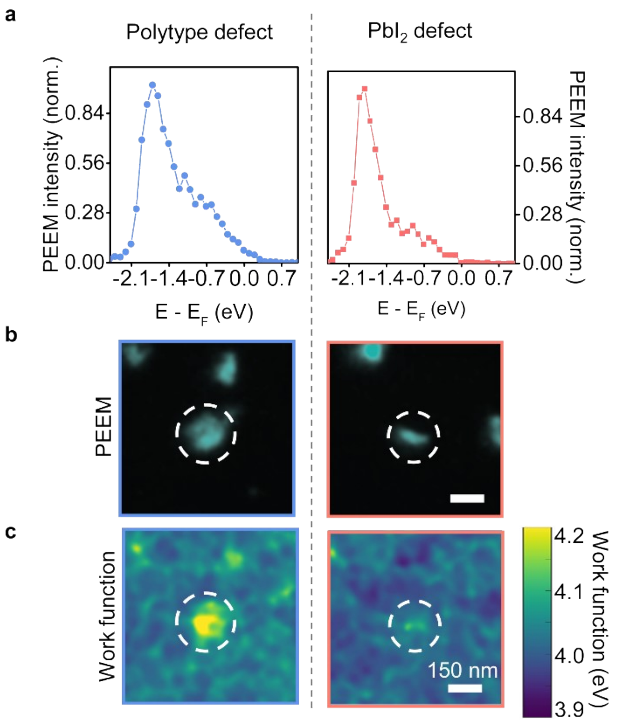

Fig. S6 Additional photoemission spectra and work function maps. (a) Photoemission spectra for polytype and PbI_2 defect clusters shown in PEEM images in (b). (c) Work function maps with nanoscale resolution for the region around the polytype and PbI_2 defect clusters shown in (b).

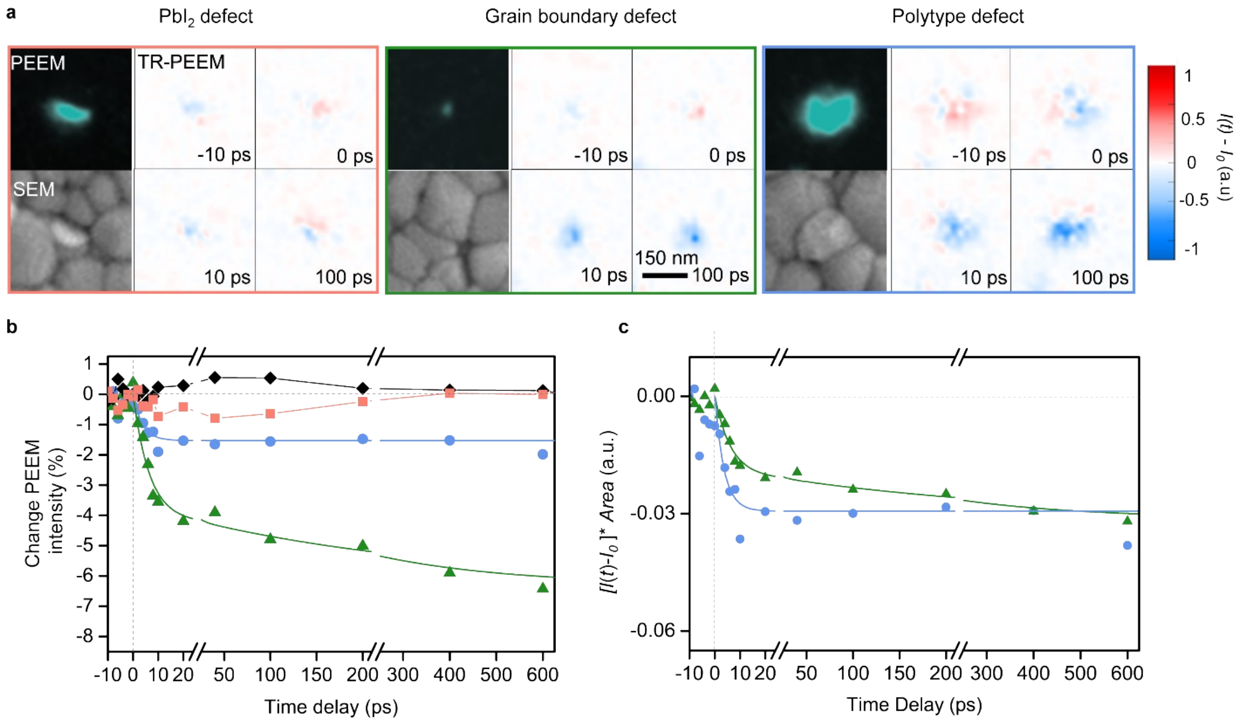

Fig. S7 Additional TR-PEEM images and traces. (a) PEEM and SEM images of three types of defect clusters with corresponding TR-PEEM intensity changes for selected pump-probe delays. I_0 indicates PEEM intensity before the photoexcitation, $I(t)$ corresponds to PEEM intensity for each time delay after the photoexcitation. (b) TR-PEEM traces indicating percentage change in PEEM intensity plotted as $[I(t) - I_0]/I_0 \times 100$ for three types of defects and region without defects. Solid lines represent bi-exponential fit for grain boundary defects (green) with time constants τ_1 = 6.4 ps ± 1.5 ps, τ_2 = 291 ps ± 162 ps and amplitudes A_1 = 4.03 ± 0.42, A_2 = 2.28 ± 0.41; and single exponential fit for polytype defects (blue) with time constant τ_1 = 3.7 ps ± 1.04 ps, and amplitude A_1 = 1.53 ± 0.08. (c) TR-PEEM traces for polytype and grain boundary defect clusters plotted as intensity change $[I(t)\text{-}I_0]\times Area$, solid lines represent single and double exponential fits.

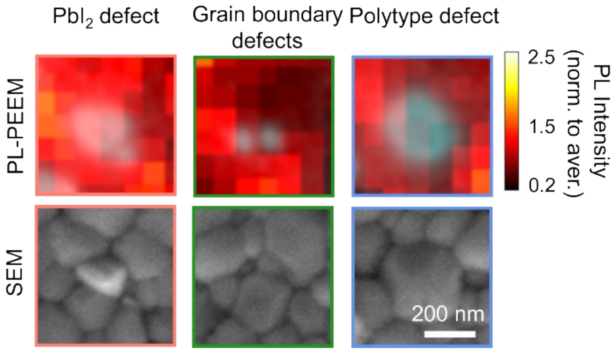

Fig. S8 Additional regions with photoluminescence (PL) maps around defect clusters (PEEM), and SEM images for selected defects. Top row shows PEEM images (green blur) overlaid on PL maps for three types of defect clusters, with corresponding SEM images (bottom row).

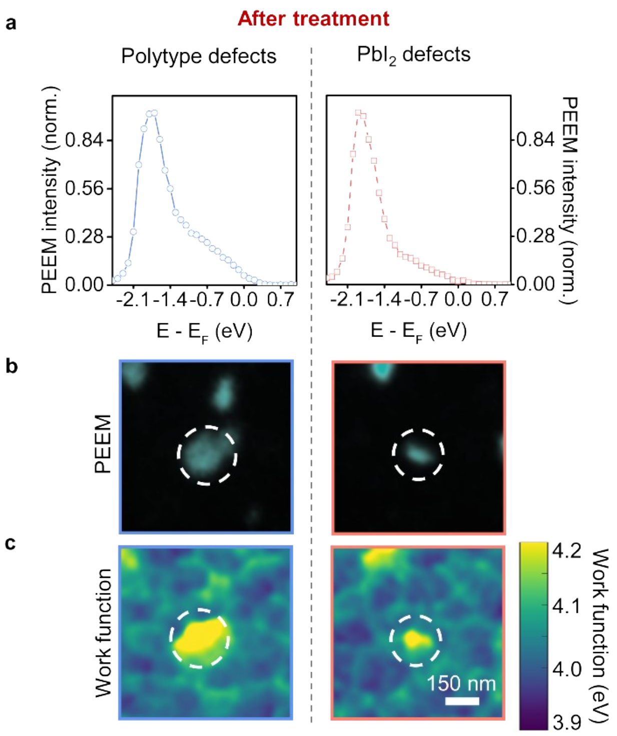

Fig. S9 Additional photoemission spectra and work function maps after treatment with dry air and light. (a) Photoemission spectra for polytype and PbI_2 defect clusters shown in PEEM images in (b), with nanoscale work function maps in **c**. While the density of mid-gap states is not largely affected after treatment with dry air and light, the work function increased for sites of PbI_2 defect clusters. Results shown for the same defect clusters as in Fig. S4.

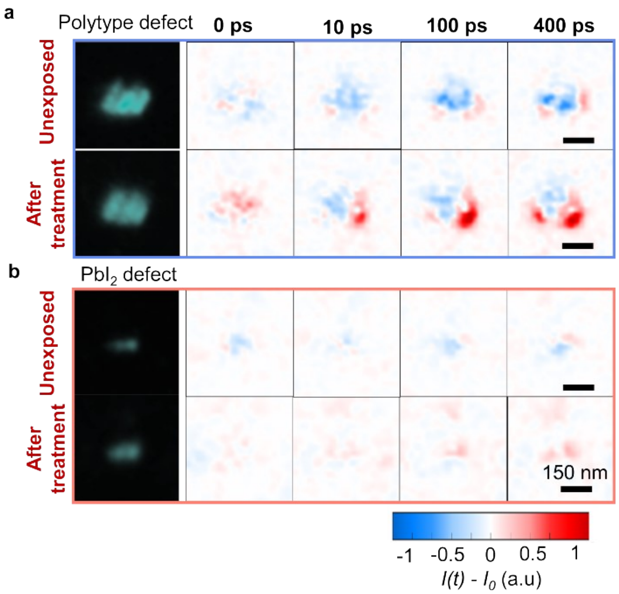

Fig. S10 TR-PEEM response of polytype and PbI_2 defect clusters upon exposure to light and dry air**.** (a) PEEM and TR-PEEM images before and after the exposure to light and dry air for polytype defect clusters. (b) PEEM and TR-PEEM images before and after the exposure to light and dry air for PbI_2 defect clusters.
